# Contribution of the non-effector members of the HrpL regulon, *iaaL* and *matE*, to the virulence of *Pseudomonas syringae* pv. tomato DC3000 in tomato plants

**DOI:** 10.1186/s12866-015-0503-8

**Published:** 2015-08-19

**Authors:** Melissa G. Castillo-Lizardo, Isabel M. Aragón, Vivian Carvajal, Isabel M. Matas, María Luisa Pérez-Bueno, María-Trinidad Gallegos, Matilde Barón, Cayo Ramos

**Affiliations:** Área de Genética, Universidad de Málaga, Instituto de Hortofruticultura Subtropical y Mediterránea “La Mayora”, Universidad de Málaga-CSIC (IHSM-UMA-CSIC), Campus de Teatinos, 29071 Málaga, Spain; German Center for Neurodegenerative Diseases, DZNE, Otfried-Müller-Straße, 27, 72076 Tübingen, Germany; Estación Experimental del Zaidín, CSIC (EEZ-CSIC), Profesor Albareda 1, 18008 Granada, Spain; Departamento de Producción Agraria, Universidad Pública de Navarra, Pamplona, Navarra Spain

## Abstract

**Background:**

The phytohormone indole-3-acetic acid (IAA) is widely distributed among plant-associated bacteria. Certain strains of the *Pseudomonas syringae* complex can further metabolize IAA into a less biologically active amino acid conjugate, 3-indole-acetyl-ε-L-lysine, through the action of the *iaaL* gene. In *P. syringae* and *Pseudomonas savastanoi* strains, the *iaaL* gene is found in synteny with an upstream gene, here called *matE*, encoding a putative MATE family transporter. In *P. syringae* pv. tomato (Pto) DC3000, a pathogen of tomato and *Arabidopsis* plants, the HrpL sigma factor controls the expression of a suite of virulence-associated genes via binding to *hrp* box promoters, including that of the *iaaL* gene. However, the significance of HrpL activation of the *iaaL* gene in the virulence of Pto DC3000 is still unclear.

**Results:**

A conserved *hrp* box motif is found upstream of the *iaaL* gene in the genomes of *P. syringae* strains. However, although the promoter region of *matE* is only conserved in genomospecies 3 of this bacterial group, we showed that this gene also belongs to the Pto DC3000 HrpL regulon. We also demonstrated that the *iaaL* gene is transcribed both independently and as part of an operon with *matE* in this pathogen. Deletion of either the *iaaL* or the *matE* gene resulted in reduced fitness and virulence of Pto DC3000 in tomato plants. In addition, we used multicolor fluorescence imaging to visualize the responses of tomato plants to wild-type Pto DC3000 and to its Δ*matE* and Δ*iaaL* mutants. Activation of secondary metabolism prior to the development of visual symptoms was observed in tomato leaves after bacterial challenges with all strains. However, the observed changes were strongest in plants challenged by the wild-type strain, indicating lower activation of secondary metabolism in plants infected with the *ΔmatE* or *ΔiaaL* mutants.

**Conclusions:**

Our results provide new evidence for the roles of non-type III effector genes belonging to the Pto DC3000 HrpL regulon in virulence.

**Electronic supplementary material:**

The online version of this article (doi:10.1186/s12866-015-0503-8) contains supplementary material, which is available to authorized users.

## Background

The ability to produce the auxin phytohormone indole-3-acetic acid (IAA) is widespread among soil and plant-associated bacteria. As more bacterial species have been analyzed, the roles of auxins in bacterial interactions with plants appear to be diverse, varying from pathogenesis to phytostimulation [1]. The best-characterized IAA biosynthetic pathway in phytopathogenic bacteria is the indole-3-acetamide pathway. In this pathway, the genetic determinants involved in the conversion of L-tryptophan (Trp) to IAA are Trp monooxygenase (encoded by the *iaaM* gene), which converts Trp to indole-3-acetamide (IAM), and IAM hydrolase (encoded by the *iaaH* gene), which catalyzes the transformation of IAM to IAA. These two genes have been cloned and characterized for phytopathogenic bacteria such as *Agrobacterium* spp. and *Pseudomonas savastanoi* [2, 3]. *P. savastanoi* pv. nerii, the causal agent of oleander (*Nerium oleander*) knot disease, also converts IAA to indole-acetyl-ε-L-lysine (IAA-Lys), a less biologically active compound as estimated in a coleoptile elongation assay [4–6]. This conversion involves the enzyme IAA-Lys synthase, encoded by the *iaaL* gene [7–9]. Although most *P. syringae* pathovars produce detectable amounts of IAA in the presence of Trp [10], IAA synthesis usually involves different genes than *iaaM* and *iaaH* [10–12]. In contrast, *iaaL* is widespread in *P. syringae* pathovars and is often found in plasmids [7, 10, 13]. Inactivation of the *iaaL* gene by transposon mutagenesis in *P. savastanoi* pv. nerii resulted in the accumulation of IAA in the culture medium; however, this mutant did not cause typical knot symptoms, probably due to its inability to multiply within host tissues [8].

*P. syringae* pv. tomato (Pto) DC3000, which causes bacterial specks on tomatoes and can infect the model plants *Arabidopsis thaliana* and *Nicotiana benthamiana*, is an important model for the study of plant-pathogen interactions [14–16]. The ability of DC3000 to infect host plants depends on numerous genes expressed by the HrpL alternative sigma factor. The Pto DC3000 HrpL regulon includes genes involved in the hypersensitive response and others encoding the pathogenicity (Hrp) type III secretion system (T3SS) machinery, a repertoire of type III effector proteins [15, 17–19], as well as genes encoding factors unrelated to the T3SS, such as *iaaL* (PSPTO_0371) and the genes for coronatine synthesis [17–21]. A recent analysis of the role of the Pto DC3000 *iaaL* gene in the infection of *N. benthamiana* plants concluded that an *iaaL* deletion mutant did not exhibit phenotypic differences in terms of *in planta* growth, virulence, or hypersensitive response (HR) compared to the wild type strain [20]. Conversely, although the genome of Pto DC3000 encodes two coding sequences (CDS) that are likely involved in auxin production [14, 22], the roles of these genes in IAA biosynthesis have not been demonstrated yet [11]. Thus, the significance of HrpL activation of the *iaaL* gene in the virulence of Pto DC3000 is still unclear.

A reporter transposon screen for HrpL-activated genes in Pto DC3000 identified several genes, including *iaaL*, that were linked to novel variations of the canonical sequence for *Hrp* boxes [17] found in HrpL-dependent promoters [23]. Further analysis of the transposon mutants revealed that the upstream promoter-proximal ORF encoded a putative MATE (multidrug and toxic compound extrusion) family transporter gene (PSPTO_0370) [17]. Recently, overexpression of HrpL in Pto DC3000 was shown to induce the expression of this putative MATE transporter gene, suggesting that it is part of the Pto DC3000 HrpL regulon [18]. Efflux pumps associated with multidrug resistance (MDR) contribute to bacterial survival in plant tissues via the removal of antimicrobial secondary metabolites, such as flavonoids, isoprenoids, and alkaloids, which are present in healthy plant tissues or synthesized *de novo* in response to pathogen attack [24, 25]. MDR efflux pumps have been shown to contribute to the colonization of host plants by bacterial phytopathogens, including *P. syringae* strains [26, 27]; however, little evidence has been found for the contribution of MATE transporters to the virulence of bacterial phytopathogens [28].

The aim of this study was to analyze the expression of the *iaaL* gene and of the putative MATE family transporter gene, hereafter called *matE*, located just upstream of *iaaL*, both in wild-type Pto DC3000 and in a knock-out Δ*hrpL* mutant generated by gene replacement. The roles of these genes in the virulence of Pto DC3000 during infection of tomato plants was analyzed, not only in terms of the pathogen-induced symptomatology observed in tomato leaves but also prior to the development of symptoms. To analyze the pre-symptomatic responses of tomato plants to bacterial infection, we used multicolor fluorescence imaging (MCFI), a technique that allows visualization of the activation of plant secondary metabolism in response to pathogen infection.

## Methods

### Bacterial strains, plasmids, and culture medium

The bacterial strains and plasmids used in this study are listed in Table 1. Pto DC3000 and mutants were routinely grown in lysogeny broth (LB) medium [29, 30] at 28 °C, and *E. coli* strains were grown at 37 °C. When appropriate, antibiotics were added to the medium at the following final concentrations (in μg/ml): for ampicillin, 100 (*E. coli* strains), 300 (Pto DC3000 strains in solid medium) and 150 (Pto DC3000 strains in liquid medium); for kanamycin, 50 (*E. coli* strains) and 25 (Pto DC3000 strains); for tetracycline, 10.

**Table 1:** Strains and plasmids used in this work

Strain/Plasmid	Genotype^a^	Reference
*P. syringae* pv. tomato		
DC3000	Rif^r^	[14]
∆*iaaL*	Km^r^	This work
∆*matE*	Km^r^	This work
∆*hrpL*	Km^r^	This work
*E. coli*		
*E. coli* DH5α	F-, ϕ*80dlacZ M15*, (*lacZYA*-*argF*) *U169*, *deoR*, *recA1*, *endA*,	[66]
	*hsdR17* (*rk* ^−^ *mk* ^−^), *phoA*, *supE44*, *thi*-*1*, *gyrA96*, *relA1*.	
Plasmids		
pGEM-T Easy Vector	Amp^r^, ori f1, *lac*Z	Promega, USA
pGEM-T Vector	Amp^r^, ori f1, *lac*Z	Promega, USA
pGEM-T-KmFRT-BamHI	Contains Km^r^ from pKD4 (Amp^r^ Km^r^)	[67]
pMP220	Expression Vector, Tc^r^	[35]
pJB3Tc20	Expression Vector, Tc^r^	[68]
pIAC1	pGEM-T derivative, contains approx. 0.5 kb on each side of the *iaaL* gene (Amp^r^)	This work
pIAC2	pGEM-T derivative, contains approx. 0.5 kb on each side of the *hrpL* gene (Amp^r^)	This work
pIAC3	pGEM-T derivative, contains approx. 0.5 kb on each side of the *mat* gene (Amp^r^)	This work
pIAC1-Km	pGEM-T derivative, contains approx. 0.5 kb on each side of the *iaaL* gene interrupted by the kanamycin resistance gene *nptII* (*Amp* ^*r*^, *Km* ^*r*^)	This work
pIAC2-Km	pGEM-T derivative, contains approx. 0.5 kb on each side of the *hrpL* gene interrupted by the kanamycin resistance gene *nptII* (Amp^r^, Km^r^)	This work
pIAC3-Km	pGEM-T derivative, contains approx. 0.5 kb on each side of the *matE* gene interrupted by the kanamycin resistance gene *nptII* (Amp^r^, Km^r^)	This work
pMP220-PmatE	pMP220 derivative, contains the *lacZ* gene expressed from the P_matE_ promoter (Tc^r^)	This work
pMP220-R2	pMP220 derivative, contains the *lacZ* gene expressed from the P_iaaL_ promoter (Tc^r^)	This work
pJB3-*matE*	pJB3 derivative, contains the *matE* gene with RBS	This work
pJB3-i*aaL*	pJB3 derivative, contains the *iaaL* gene with RBS	This work

^a^Amp^r^, Km^r^, Rif^r^ and Tc^r^ indicate resistance to ampicillin, kanamycin, rifampicin and tetracycline, respectively

### Preparation of total RNA

RNA was extracted from cultures grown on M9 minimal media supplemented with 5 mM mannitol [30, 31] and ferric citrate at 0.0006 %. The cells were pelleted at exponential phase, i.e., when they reached an optical density of 0.5 at 660 nm (OD_660nm_). The pellets were processed for RNA isolation using TriPure Isolation Reagent (Roche Applied Science; Mannheim, Germany) according to the manufacturer’s instructions with the following exceptions: the TRIPure was preheated to 65 °C, the lysis step was carried out at 65 °C and BCP (1-bromo-3-chloropropane) (Molecular Research Center; Cincinnati, OH, U.S.A.) was used instead of chloroform. The RNA concentration was determined spectrophotometrically, and its integrity was assessed by agarose gel electrophoresis. Total RNA was treated with a TURBO DNA-*free*™-Kit (Applied Biosystems; California, U.S.A.) as detailed by the manufacturer. Subsequently, the samples were tested for genomic contamination by PCR.

### Primer extension analysis

DNA-free RNA, prepared as described above, was heat-treated at 80 °C for 5 min. Subsequently, 30 μg of RNA was annealed at 75 °C for 10 min, and then the temperature was allowed to slowly decrease to 60 °C within 30 min. Annealing was performed in a buffer containing 100 mM NaCl and 50 mM Tris-Cl at pH 7.5. The specific primers (Additional file 4: Table S1) and the φX174 DNA/*Hinf*I dephosphorylated marker were labeled using [γ^32^-P]ATP (3000 Ci/mmol) (PerkinElmer; Boston, USA) and T4 polynucleotide kinase, according to the manufacturer’s instructions for the Primer Extension System-AMV Reverse Transcriptase (Promega; Madison, USA). Runoff reverse transcription reactions were performed for 1 h at 60 °C using 15 units of ThermoScript™ RNase H- (Invitrogen; California, USA) in its provided buffer (complemented with 1 mM of each dNTP and 5 mM DTT). Reactions were stopped by the addition of one volume of loading dye (Promega; Madison, USA) and were analyzed on 6 % polyacrylamide sequencing gels containing 8 M urea. The results were visualized either using X-ray films or exposure for 24 h to Imaging Plates (IP BAS-MP 2040S), which were analyzed with a Fujifilm-BAS 1500 (Fuji; Tokyo, Japan).

### Transcription initiation mapping by 5**′**cRACE

The transcription start site of *matE* was determined using the 5′cRACE method [32–34]. cDNA synthesis was performed using total DNA-free RNA, obtained as described above. One microgram of this RNA was used as a template to synthesize first-strand cDNA using a SMART^™ ^ RACE cDNA Amplification synthesis kit (Clontech; California, USA), and a gene-specific primer was designed to anneal within the coding region of the gene (Additional file 4: Table S1). The reactions proceeded for 90 min at 42 °C. Then, they were diluted 10-fold in water, and 1 μl of these dilutions were used as templates in 20-μl PCR reactions. The PCR cycles were as follows: 5 cycles for 30 s at 94 °C; 3 min at 72 °C; 5 cycles for 30 s at 94 °C; 30 s at 70 °C; 3 min at 72 °C; 25 cycles for 30 s at 94 °C; 30 s at 68 °C; 3 min at 72 °C. The amplification products were cloned into the pGEM®-T Easy Vector (Promega; Madison, USA) and sequenced.

### RT-PCR

DNA-free RNA was reverse transcribed using random hexamers included in the iScript™ cDNA synthesis kit (BioRad; California, USA). Afterwards, PCR reactions were performed with GoTaq® polymerase (Promega; Madison, USA) using 100 ng of cDNA as a template and specific primers detailed in Additional file 4: Table S1. Products were analyzed by agarose gel electrophoresis.

### β-galactosidase activity assays

β-galactosidase activity was measured from DNA fragments cloned into the expression vector pMP220 [35] using the methods developed [29] and modified previously [36]. In summary, cells carrying the plasmids were cultured in LB media supplemented with 10 μg/ml tetracycline. Grown cultures were harvested by centrifugation and washed three times with 0.9 % NaCl. Finally, cultures were adjusted to an OD_600nm_ of 0.5 and cultured in minimal media as described above [30, 31].

To measure β-galactosidase activity, 200 μl of each culture were lysed with 100 μl of chloroform and 50 μl of 0.1 % SDS and were then mixed with 800 μl of Z buffer (40 mM Na_2_HPO_4_, 40 mM NaH_2_PO_4_, 10 mM KCl, pH 7.0 and 50 mM β-mercaptoethanol, added just before use). After mixing, samples were incubated at room temperature for 5 min. Then, 200 μl of Z buffer containing 4 mg/ml o-nitrophenyl-β-D-galactopyranoside (ONPG) were added. The samples were then incubated at room temperature for 30–60 min until they turned yellow. Afterwards, reactions were stopped with 500 μl of 1 M Na_2_CO_3_, and the absorbance was measured at 420 and 550 nm. In addition, absorbance at 600 nm was measured in the initial culture. The enzymatic activity (in Miller units) was determined with the equation [(A_420_ − 1.7*A_550_)*1000]/(V*A_600_*t) [29], where A_XXX_ is the absorbance at 420, 550 and 600 nm, V is the volume of the culture in ml and t is the time of the reaction in minutes.

### Generation of knockout mutants

Plasmids pIAC1, pIAC2, and pIAC3 were generated to obtain Δ*iaaL*, Δ*hprL*, and Δ*matE* mutant strains, respectively. DNA fragments of approximately 0.5 kb corresponding to 5′ and 3′ regions flanking the target gene were cloned into the pGEM-T Easy vector (Promega; Madison, USA). This fragment was generated with three rounds of PCR amplification using the method described by [37]. All plasmids were sequenced to verify the absence of mutations.

Following sequencing, plasmids were labeled with the *nptII* kanamycin resistance gene flanked by FRT (flippase recognition target) sites. The fragment containing this gene sequence was obtained from the plasmids pGEM-T-KmFRT-BamHI or pGEM-T-KmFRT-EcoRI (Table 1). The fragments resulting from digestion were cloned into the BamHI or EcoRI sites of vectors derived from pGEM-T containing previously amplified fragments.

Plasmids pIAC1, pIAC2 and pIAC3 (Table 1) were transformed by electroporation into the Pto DC3000 strain. Transformants were selected on LB media supplemented with kanamycin (15 μg/ml). Replica plates of the resulting colonies were created in plates with ampicillin (300 μg/ml) to determine whether each transformant was the result of a single recombination event (integration plasmid, Amp^R^) or a double recombination event (allelic exchange, Amp^S^). Southern Blot analysis using a sequence complementary to a target gene as a probe was used to confirm whether allelic exchange had occurred in the correct location of the genome and that only one copy of the construct was inserted.

### Virulence assays and symptom quantification in tomato plants

Seeds of tomato plants (*Solanum lycopersicum* var. Moneymaker) were germinated and grown in a growth chamber with a 16/8 h light/dark photoperiod at 24/18 °C day/night and at 70 % relative humidity. Bacteria were grown on LB agar plates for 48 h at 28 °C and resuspended in 10 mM MgCl_2_ at an OD_600nm_ of 0.5, corresponding to about 10^8^ colony forming units (CFU) per ml. Further serial dilutions were carried out to obtain suspensions for inoculations with different doses. Four to 5-week-old plants were inoculated by infiltrating bacterial suspensions into the intracellular spaces. Infiltration was achieved by pressing the bacterial suspension against the leaf with a 2-ml syringe without the needle. The negative control plants were mock-inoculated with a 10 mM MgCl_2_ solution. Each plant was infiltrated in three leaflets of the same leaf, and the infiltrated (IF) area covered half of the midrib on the right side of the main vein. The other halves of those leaflets were analyzed as non-infiltrated (NIF) areas. The evolution of disease symptoms was recorded at 2, 4, 7 and 9 days post-inoculation (dpi). Bacteria were recovered from the infected leaves using a 10-mm diameter cork borer. Five disks (3.9 cm^2^) per plant were homogenized via mechanical disruption in 1 ml of 10 mM MgCl_2_, and the CFU per cm^2^ were counted by plating serial dilutions on LB plates with corresponding antibiotics. Quantification of necrotic lesions was performed at 9 dpi using the image analysis software Visilog 6.3 (Noesis; Courtaboeuf, France). Data were represented as the ratios of necrotic lesions per total leaf area.

### Competition assays in tomato plants

Competition assays were performed by mixing cultures of mutants and wild-type strains with an OD_660nm_ of 0.5 in a 1:1 ratio. Four to 5-week-old tomato plants were inoculated with 5 × 10^4^ CFU/ml mixed bacterial suspensions using a 2-ml syringe without the needle. Serial dilutions of the inocula were plated in LB with and without 15 μg/ml kanamycin for the selection of mutant and wild-type strains, respectively. Four days post-inoculation, bacteria were recovered from the infected leaves by grinding the tissue with 1 ml of 10 mM MgCl_2_, and the CFU per cm^2^ were counted by plating serial dilutions on LB plates amended with the corresponding antibiotics. A competitive index (CI) was calculated by dividing the output ratio (CFU mutant:CFU wild-type) by the input ratio (CFU mutant:CFU wild-type). The competition indices shown are the means of three replicates showing typical results from two independent experiments, i.e. six replicates in total. Statistical analysis of CI values was carried out using Student’s *t* test and the hypothesis that the mean index was not significantly different from 1.0 (*P*-value = 0.005).

### Fluorescence imaging

Fluorescence images of the adaxial (AD) surfaces of leaves were captured with the customized fluorescence imaging system Open FluorCam FC 800-O (Photon Systems Instruments, Brno, Czech Republic). Autofluorescence emission of the leaves was excited with a UV source (360 nm), and the F440, F520 and F690 images were acquired sequentially from identical fields of view according to [38]. Black and white images of both measured fluorescence intensity and the calculated fluorescence ratio (F440⁄F690) are shown using a false color scale. Images and numerical data from regions of interest were processed with FluorCam software version 7.1.0.3 (PSI Systems, Brno, Czech Republic). Measurements were carried out in mock-control and bacterial-challenged plants at 2, 4, 7, and 9 dpi. Four plants per treatment were analyzed, and the experiment was carried out three times with similar results.

## Results and discussion

### Genomic context analysis of the *iaaL* and *matE* loci in *P. syringae* and *P. savastanoi*

**Fig. 1:**
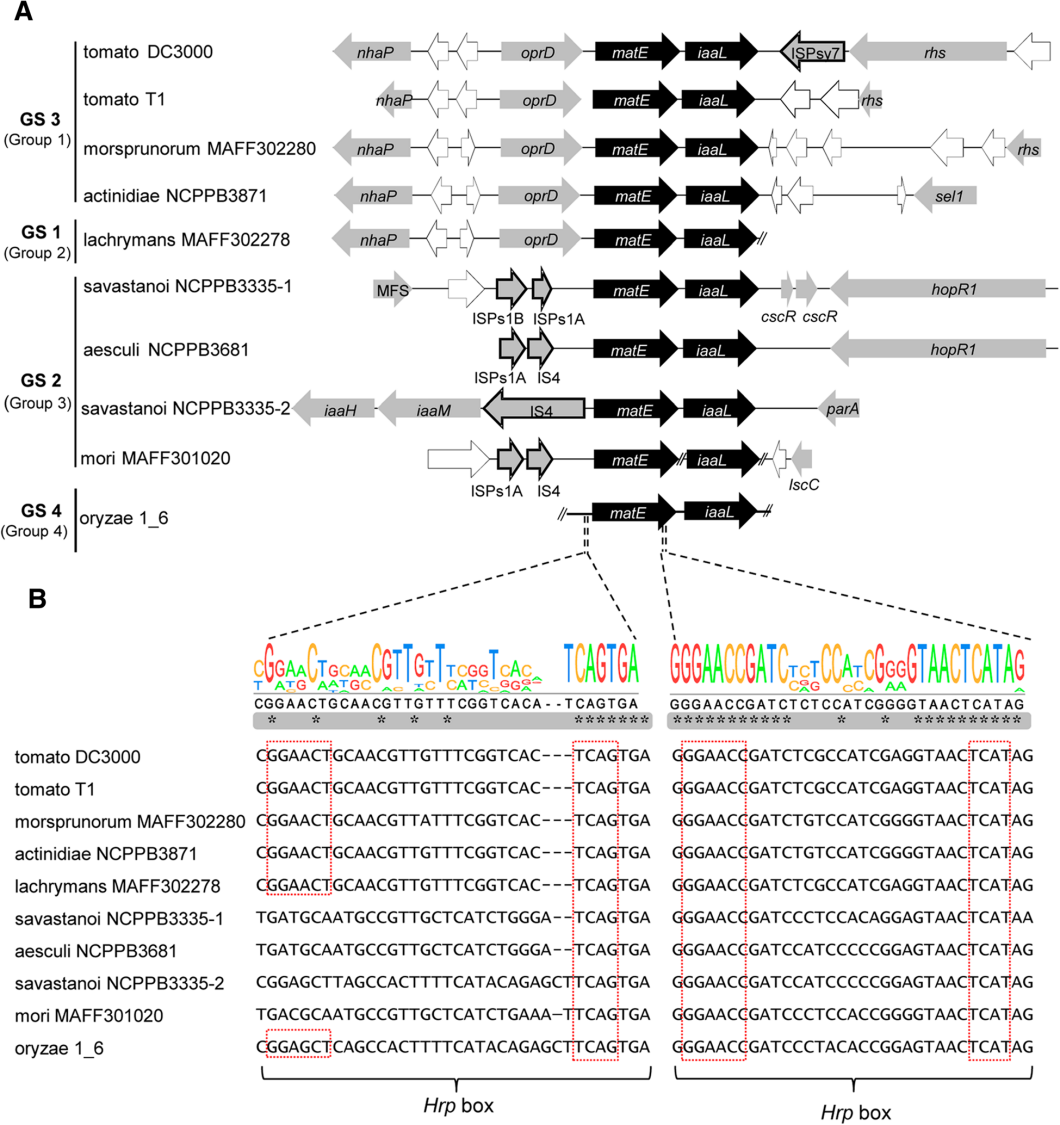
Bioinformatics analysis of the *iaaL* and *matE* genes in the *P. syringae* complex. **a** Gene organization of the *matE* and *iaaL* loci in nine strains of the *P. syringae* complex. *Arrows* indicate the direction of transcription and the relative sizes of the genes in the genome of the indicated strains except for the hopR1 gene, whose size is longer than that shown in this figure. *White arrows*, genes encoding hypothetical proteins; *gray arrows*, genes with known functions. *GS* genomospecies [62]; *IS* insertion sequence (*bold*-*lined gray arrows*); Group, Multi-Locus Sequence Typing (MLST) groups [22, 39]. *Double bars* indicate the end of a contig in the corresponding draft genome. The *P. syringae* groups 1, 3 and 4 [39, 63, 64] correspond to genomospecies (GS) 3, 2 and 4, respectively. **b** Multiple nucleotide sequence alignment of *matE* and *iaaL* putative promoters. *Asterisks* indicate nucleotides conserved in all strains analyzed. DNA sequences were downloaded from the ASAP database (http://www.genome.wisc.edu/tools/asap.htm). *Red squares* show the consensus motif of the *hrp* box sequences. −1 and −2 indicate the two different *matE* alleles encoded in the genome of *P. savastanoi* pv. savastanoi NCPPB3335, AER-0005392 and AER-0005391 [65], respectively

Gene *iaaL* is widely distributed within the *P. syringae* complex, mainly within genomospecies 2, 3 and 4 corresponding to phylogenetic Multi-Locus Sequence Typing (MLST) groups 3, 1 and 4, respectively [22, 39]. Conversely, the *iaaL* phylogeny is largely congruent with that of the *P. syringae* complex deduced from housekeeping genes, suggesting that *iaaL* is ancestral to the complex. Furthermore, several copies of *iaaL* are often found in close proximity to insertion sequences or encoded in plasmids, all of which provide evidence of horizontal transfer [11, 40]. Genomic context analysis of the *iaaL* gene in a selection of sequenced *P. syringae* and *P. savastanoi* strains belonging to genomospecies 2, 3 and 4 revealed that this gene is under synteny with the *matE* gene in all of these strains (Fig. 1a). Bioinformatics prediction of transmembrane helices in the Pto DC3000 MatE protein using the TMHMM Server v.2.0 showed a distribution of 12 putative transmembrane helical regions (Additional file 1: Figure S1) characteristic of the MATE family transporters [28, 41]. Furthermore, several ORFs were conserved in presence and arrangement in all strains belonging to genomospecies 3, e.g., *P. syringae* pv. lachrymans MAFF302278 and Pto DC3000. These ORFs encode for an outer membrane porin (*oprD*), an Na^+^/H^+^ antiporter (*nhaP*) and two hypothetical proteins. In contrast, strains belonging to genomospecies 2 demonstrated a completely different genomic context, both among each other and in comparison with genomospecies 3. In agreement with the suggested horizontal transfer of the *iaaL* gene among strains of the *P. syringae* complex [11], insertion sequence elements were found upstream of the *matE* gene in the genomes of all genomospecies 2 strains analyzed (Fig. 1a). A role for these insertion sequences in the dissemination and evolution of IAA-related genes was previously suggested by other authors [42, 43]. Moreover, synteny was not maintained in the downstream region of the *matE*-*iaaL* loci for any of the genomes analyzed (Fig. 1a), further supporting the possible integration of a mobile *matE*-*iaaL* cassette at different genome locations during the evolution of this bacterial group.

The existence of *hrp* box promoter sequences upstream of both the *iaaL* and *matE* genes has been reported in Pto DC3000 [17–20]. However, these two *hrp* box motifs were defined as non-canonical [17, 18, 20]. Although polymorphisms in *hrp* box sequences have been correlated with the loss of HrpL-dependent expression [18], the divergent *hrp* box promoter of the *iaaL* gene [19] actually binds HrpL and promotes HrpL-dependent transcription [20]. Interestingly, the promoter sequence of the *iaaL* gene is encoded within the 3′ end of the *matE* gene in Pto DC3000 (Fig. 1b), suggesting that it could be constrained by the genetic code. Indeed, multi-sequence alignment of the *iaaL* promoter showed a conserved 5′-GGAACC-N_20_-TCAT-3′ motif in the genomes of all *P. syringae* strains analyzed, including members of genomospecies 2, 3 and 4 (Fig. 1b). Other *hrp* box promoters found embedded within their upstream encoded ORFs include PSPTO_2130 and its orthologs, which are also members of the non-effector genes included in the *P. syringae* HrpL regulon [18].

In terms of the promoter region of *matE*, a highly conserved *hrp* box motif, 5′-GGAACT-N_19_-TCAG-3′, was found in the strains of genomospecies 3 and exhibited a slight variation in its −35 box (5′-GGAGCT-3′) in *P. syringae* pv. oryzae (Por) 1_6 (genomospecies 4, MLST group 4). However, although the putative −10 region of this promoter (5′-TCAG-3′) was also conserved in all strains included in genomospecies 2, a consensus −35 region was not found in the genomes analyzed (Fig. 1b), suggesting a possible loss of HrpL regulation of the *matE* promoter in this genomospecies. Transcription of the *matE* gene has been shown to be upregulated in response to HrpL overexpression both in Pto DC3000 (genomospecies 3) and Por 1_6 (genomospecies 4) [18], suggesting that the polymorphisms observed between these genomospecies in the −35 *hrp* box of *matE* do not seem to alter HrpL-dependent transcription of this gene. Low conservation of the −35 region has been observed for other promoters regulated by extracytoplasmic function (ECF) sigma factors, of which HrpL is an example [44, 45], and also for RpoD-dependent promoters [46, 47], However, downregulation of *matE* in a Δ*hrpL* background has not yet been reported for any *P. syringae* strain.

### The *iaaL* gene is transcribed both independently and in formation in an operon with the *matE* gene

RNA-Seq combined with Illumina high-throughput sequencing technology have been used to identify 5′-ends of transcripts in Pto DC3000 grown in iron-limited MG medium, including those corresponding to the *iaaL* gene [33, 34]. Moreover, transcriptome profiling using RNA-seq coupled with the GENE-counter software package [48] recently expanded characterization of the HrpL regulon from six *P. syringae* strains, including Pto DC3000, and pointed again at the *iaaL* and *matE* genes as members of this regulon [18]. Moreover, chromatin immunoprecipitation coupled with high-throughput sequencing (ChIP-Seq) and RNA-Seq was used to identify HrpL-binding sites and likely *hrp* promoters, including that of the *iaaL* gene [20]. Despite the growing use of RNA-Seq as a high-throughput strategy to analyze the transcriptome of Pto DC3000 on a global scale, very few confirmed transcriptional start sites have been reported for this model pathogen [34]. With the experimental aim of confirming the transcription start sites of both the *iaaL* and *matE* genes in Pto DC3000, we first used primer extension analysis and then 5′RACE. The transcription start site of *iaaL* was located just three nucleotides downstream of the 3′ end of its proximal *hrp* box promoter, at the C located 79 bp upstream of its start codon (Additional file 2: Figure S2). This position is placed two nucleotides upstream and one nucleotide downstream, respectively, of those previously determined [20, 34]. Extension of several primers complementary to the *matE* sequence yielded no amplification products. However, the transcription initiation site of the *matE* gene was identified by 5′RACE at the G located 66 bp upstream of its start codon (Additional file 2: Figure S2). This start site is identical to one of the two possible sites proposed by [34] and is located 17 bp downstream of the 3′ end of its proximal *hrp* box promoter (Fig. 1). A detailed analysis of the transcription start sites of HrpL-regulated promoters determined in Pto DC3000 by [34] revealed that most of them, 39 of the 49 identified, were located at a distance within 2–4 bp to the proximal *hrp* box promoter; the remaining distances from the start sites ranged from 0 to 75 bp (Additional file 3: Figure S3).

**Fig. 2:**
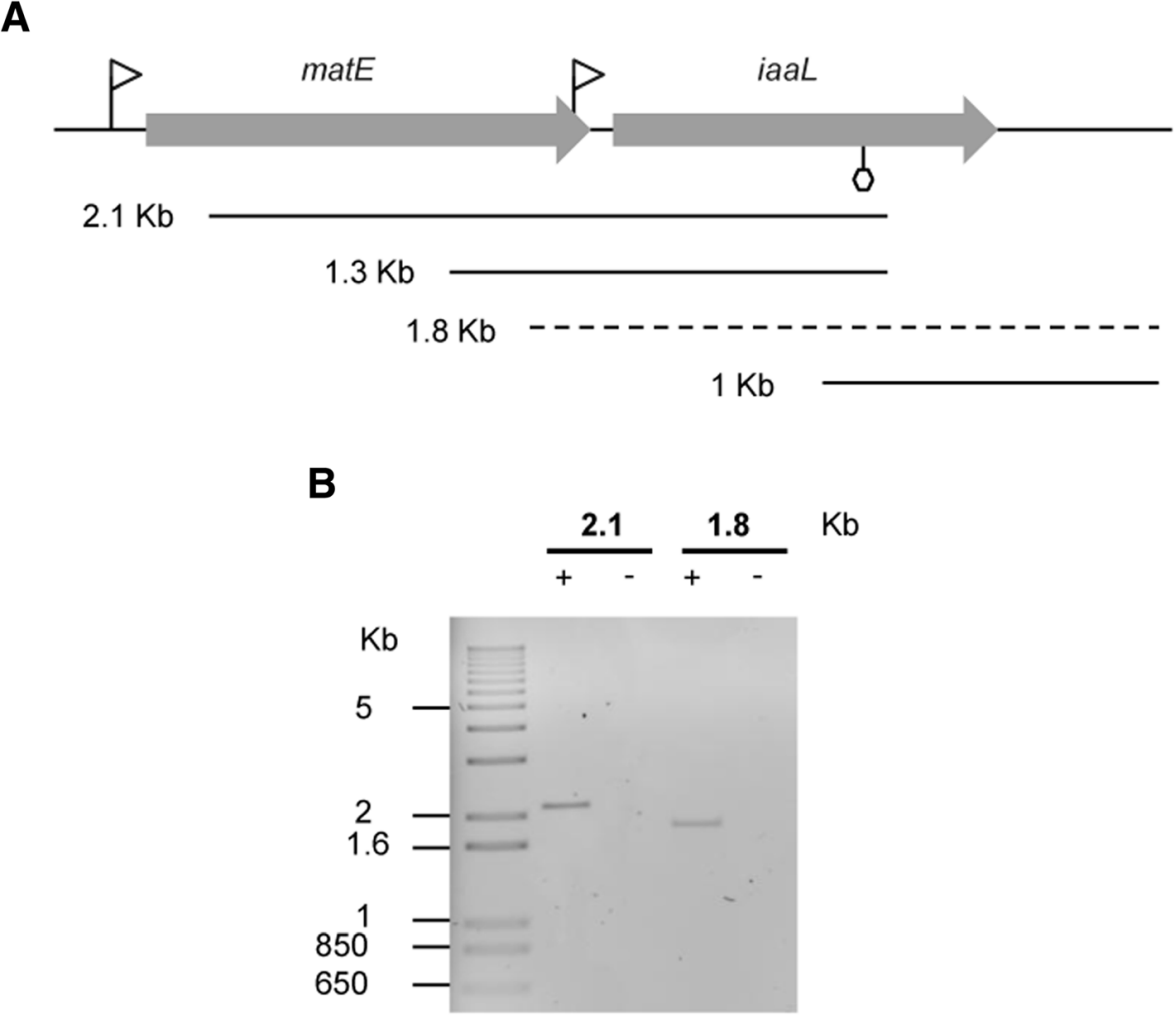
Co-transcription of the *P. syringae* pv. tomato DC3000 *matE* and *iaaL* genes. **a** A diagram of the *matE* and *iaaL genes* is shown. The direction of transcription is indicated by the *arrows. Triangles* represent *hrp* box promoters, and the *hexagon* shows a putative transcriptional terminator predicted using a web interface [50]. *Solid* and *dashed lines* represent RT-PCR products. Sequenced products are indicated by *solid lines*. **b** Agarose gel electrophoresis of RT-PCR products encoding the intergenic regions of the *matE*-*iaaL* operon. The sizes of the products are indicated on *top* of the lanes. + and − indicate reaction mixtures containing or lacking Pto DC3000 c-DNA, respectively. At the *left*, a 1-Kb molecular ladder is shown

The existence of a *matE*-*iaaL* operon has been suggested in both Pto DC3000 [18–20] and Pto T1 [49]. However, co-transcription of these two genes has not been experimentally demonstrated to date. To further explore this hypothesis, RT-PCR analyses were performed using total RNA extracted from Pto DC3000 together with forward and reverse primers (Additional file 4: Table S1) annealing within the *iaaL* and *matE* gene sequences, respectively (Fig. 2a). A product of approximately 2.1 Kb, including most of the *matE* ORF, the intergenic region between *matE* and *iaaL* and more than 70 % the *iaaL* ORF, was obtained and confirmed by sequencing. A putative rho-independent transcriptional terminator was located using a web interface [50] near the 3′ end of this amplicon (Fig. 2b). However, a product of approximately 1.8 Kb spanning from the 3′ end of the *matE* gene to 196 bp downstream of the stop codon of *iaaL* was also obtained (Fig. 2b), further supporting the notion of the co-transcription of *matE* and *iaaL*. Together, all of these results demonstrate that the Pto DC3000 *iaaL* gene can be transcribed both independently and as an operon with the *matE* gene.

### Transcription from the *P. syringae* pv. tomato DC3000 P_*matE*_ promoter is HrpL-dependent

**Fig. 3:**
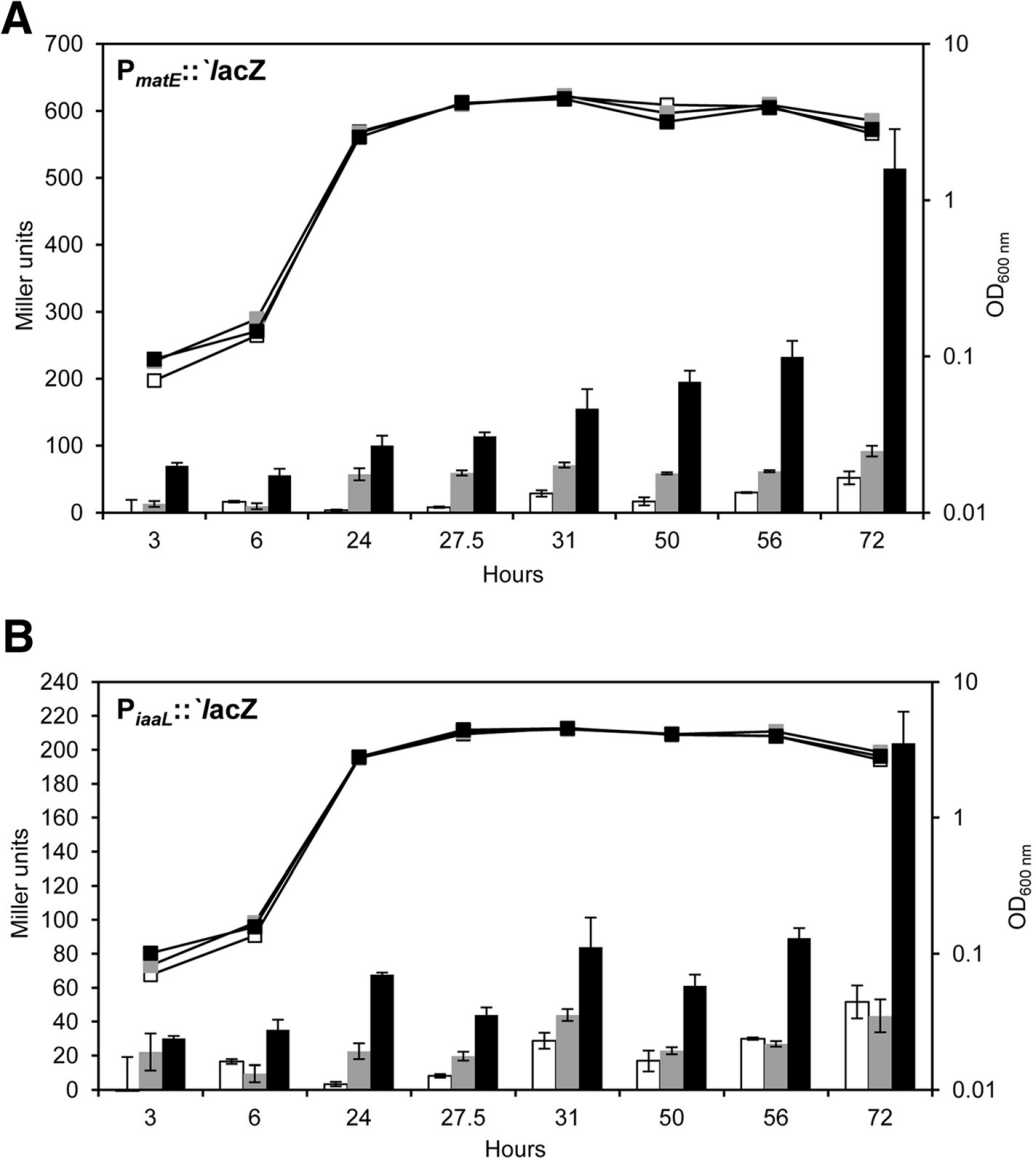
HrpL-dependent expression of the *matE* (P_*matE*_) and *iaaL* (P_*iaaL*_) promoters in *P. syringae* pv. tomato (Pto) DC3000. Growth curves of wild-type Pto DC3000 transformed with the empty vector pMP220 (*white*) or with a vector carrying the promoter fusions P_*matE*_::`*l*acZ (**a**) or P_*iaaL*_::`*l*acZ (**b**) (*black*). *Bars* show the β-galactosidase activity obtained from P_*matE*_::`*l*acZ (**a**) or P_*iaaL*_::`*l*acZ (**b**) during the growth of Pto DC3000 derivatives on minimal M9 medium. Pto∆*hrpL*, ∆*hrpL* Pto DC3000 mutant (*grey*) carrying P_*matE*_::`*l*acZ (**a**) or P_*iaaL*_::`*l*acZ (**b**). The results shown are the means of three different experiments with their respective standard deviations

To unveil the HrpL-dependency of the expression of the *matE* gene, a Δ*hrpL* Pto DC3000 mutant was constructed. To analyze the in vivo activity of the predicted promoter region of *matE* (P_*matE*_), a transcriptional fusion of this promoter to *lacZ* was constructed and transformed into both the wild-type strain Pto DC3000 and its Δ*hrpL* mutant. In addition, Pto strains expressing a fusion of the *iaaL* promoter (P_*iaaL*_) to *lacZ* were also constructed for comparison. A Pto DC3000 transformant carrying the plasmid pMP220 (empty vector) was used as a negative control. In the wild-type strain, the activity of P_*matE*_ increased along the growth curve, reaching a maximal expression of over 500 Miller units after 72 h (late stationary phase). Moreover, this activity was clearly higher than that found for the Δ*hrpL* mutant, whose activity remained substantially consistent throughout the growth curve, and higher than that observed for Pto DC3000 carrying the empty vector (Fig. 3a). Thus, our results show that the expression from P_*matE*_ in Pto DC3000 may take place from two different promoters: one weak and constitutive and another stronger and HrpL-dependent. With the exception of *P. syringae* pv. morsprunorum (Pmp) MAFF302280, all other *P. syringae* strains belonging to genomospecies 1 and 3 analyzed in this study encode a P_*matE*_ promoter sequence identical to that of Pto DC3000 (Fig. 1b). Thus, it could be expected that the expression of *matE* in all these strains is also activated by HrpL. Polymorphisms in the consensus *hrp* box promoter sequence have been shown to be responsible for the loss of HrpL-dependent transcription of other none-effector genes in Pto DC3000, i.e., the promoter sequences of PSPTO_2130 and PSPTO_2105 [18]. Therefore, the polymorphisms observed in the promoter sequences of P_*matE*_ in Pmp MAFF302280 and in the strains of *P. syringae* and *P. savastanoi* included in genomospecies 1, 2 and 4 (Fig. 1b) might be responsible for the recruitment of the *matE* gene to the HrpL regulon in all of these strains. However, this hypothesis has yet to be tested.

The activity of the P_*iaaL*_ promoter increased after 6 h and remained moderately stable from 24 to 56 h, reaching a maximum level of approximately 200 Miller units after 72 h. In agreement with other authors [20], the activity of this promoter was fully dependent on HrpL, as ß-galactosidase activity levels obtained in the Δ*hrpL* mutant were generally similar to those of the negative control (Fig. 3b). In summary, expression from both P_*matE*_ and P_*iaaL*_ is dependent on HrpL and is induced in late stationary phase, although the induction levels of P_*matE*_ are approximately 2.5 times higher than P_*iaaL*_ under the conditions tested. Although analysis of *P. syringae* virulence has primarily focused on the characterization and function of the T3SS and its effector proteins, a role in virulence has also been demonstrated for other non-effector genes regulated by HrpL, e.g., the Pto DC300 *corR* [17, 51] and *aprI* [20, 52] genes and the PSPPH_A0106-A0112 operon of *P. syringae* pv. phaseolicola 1448A [18]. In the light of these findings, the possible roles of the *matE* and *iaaL* genes in the survival and virulence of Pto DC3000 in tomato plants were analyzed.

### The *iaaL* and *matE* genes are required for full virulence of *P. syringae* pv. tomato DC3000 in tomato plants

**Fig. 4:**
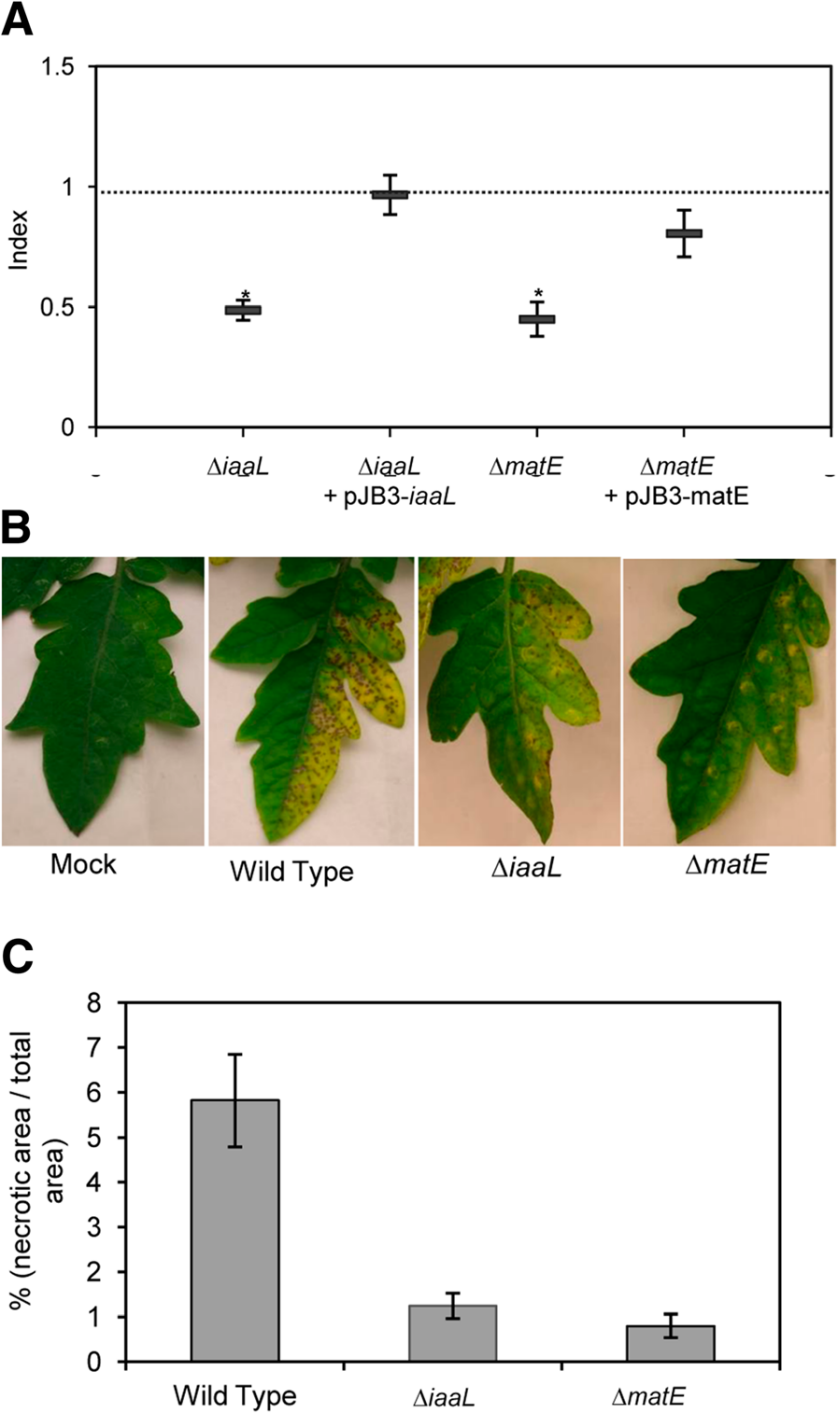
Hypovirulence of *P. syringae* pv. tomato DC3000 *iaaL* and *matE* mutants in tomato plants. **a** Competitive index (CI) values for mixed inoculations of *P. syringae* pv. tomato DC3000 and its derivate strains in tomato plants. Complementation was performed via ectopic expression of *iaaL* or *matE* cloned in the expression vector pJB3 (Table 1). *Error bars* indicate the standard error from the average of two different assays. *Asterisks* indicate CI values that were significantly different from one. Statistical analyses were performed using Student’s *t*-test and P = 0.005. **b** Representative images of symptoms induced by the indicated strains in var. Moneymaker tomato plants at 9 days post-inoculation (dpi). Mock, plants inoculated with a MgCl_2_ solution. **c** Quantification of necrotic areas in the leaflets shown in (b). The results are the means of nine different leaflets. *Error bars* represent the standard error

To analyze the roles of the *iaaL* and *matE* genes in the fitness and virulence of Pto DC3000, knock-out mutants of each of these genes and their corresponding complemented strains were constructed. While the Δ*iaaL* mutant carried a deletion of the complete *iaaL* ORF, the Δ*matE* mutant encoded a deletion expanding from the start codon of the gene to 61 bp upstream of its 3′ end. Thus, the Δ*matE* deletion did not affect the *hrp* box of P_*iaaL*_, which is embedded in the 3′ end of *matE* (Fig. 1). Competition assays between each of the mutants, or their corresponding complemented strains, against the wild-type strain Pto DC3000 were carried out. Tomato leaves were infiltrated with mixed inocula (1:1), and after 5 days, bacteria were recovered and viable cells were counted. The results shown in Fig. 4a are expressed as competition indices (CI) of the mutants (Δ*iaaL* and Δ*matE*) or complemented strains (Δ*iaaL* pJB3-*iaaL* and Δ*matE* pJB3-*matE*) relative to the wild-type strain. The ability of both mutant strains to multiply and survive *in planta* was clearly impaired in comparison with the wild-type strain, as reflected by the CI values of approximately 0.5 obtained for each of the mutants. However, ectopic expression of the *iaaL* or *matE* genes in their corresponding mutant strains yielded CI values that were not significantly different from one (Fig. 4a), demonstrating full complementation of the growth defects of the mutants via plasmid-borne expression of the deleted genes. Under the same conditions used in this study, CI assays have previously permitted the detection of virulence phenotypes for Pto DC3000 genes, including T3SS effectors proven to be functionally redundant [53]. Thus, our results demonstrate that the *matE* and *iaaL* genes are both required for growth and survival of Pto DC3000 in tomato leaves. Virulence of bacterial pathogens in host plants, including Pto DC3000 [15], is often dependent on their ability to grow and survive in plant tissues. Therefore, we decided to test the ability of the Pto DC3000 Δ*iaaL* and Δ*matE* mutants to cause disease in tomato leaves.

Figure 4b shows the symptoms in tomato leaves caused by wild-type Pto DC3000 and the Δ*iaaL* and Δ*matE* mutants at 9 dpi. Moreover, image analysis of infected leaves was also performed at 9 dpi, allowing the quantification of the symptoms induced as the ratio of necrotic lesions/total leaf area (Fig. 4c). At 9 dpi, chlorosis of the tissue induced by wild-type Pto DC3000 covered the entire inoculated area, and the necrotic lesions covered approximately 6 % of the total leaf area. In contrast, leaves inoculated with the Δ*matE* or Δ*iaaL* mutants developed late chlorosis and exhibited significantly fewer necrotic lesions than those caused by the wild-type strain (approximately 0.8 and 1.2 % of the total leaf area, respectively) (Fig. 4b and c). Therefore, both *matE* and *iaaL* are needed for growth and fitness of Pto DC3000 in tomato leaves, which also have an effect on its virulence.

It has been recently studied whether some of the non-effector genes identified as new members of the Pto DC3000 HrpL regulon contributed to pathogenicity. For that, deletion mutants were constructed (on wild-type Pto DC3000 and Δ*hopQ1*-*1* backgrounds) for seven candidates, including the *iaaL* gene. Despite the fact that the annotated functions of all seven genes were elusive in terms of plant association, no phenotypic differences were observed for in planta growth, virulence, or HR for any of these mutants in *Nicotiana benthamiana* plants [20]. In addition to differences in the experimental design of these two pathogenicity tests, the *iaaL* gene may have a relevant role in the interaction of this pathogen with tomato plants and not with *N. benthamiana*, a host plant for a mutant lacking the T3SS effector HopQ1-1 but not for wild-type Pto DC3000 [54]. On the other hand, MatE most likely contributes to the intrinsic bacterial resistance to toxic compound(s) produced by tomato plants, thus promoting Pto DC3000 survival during infection. Since the *matE* gene was shown to be induced *in planta* [21], the contribution of MatE to bacterial resistance might be emphasized in this case.

### Multicolor fluorescence imaging of infected tomato plants

**Fig. 5:**
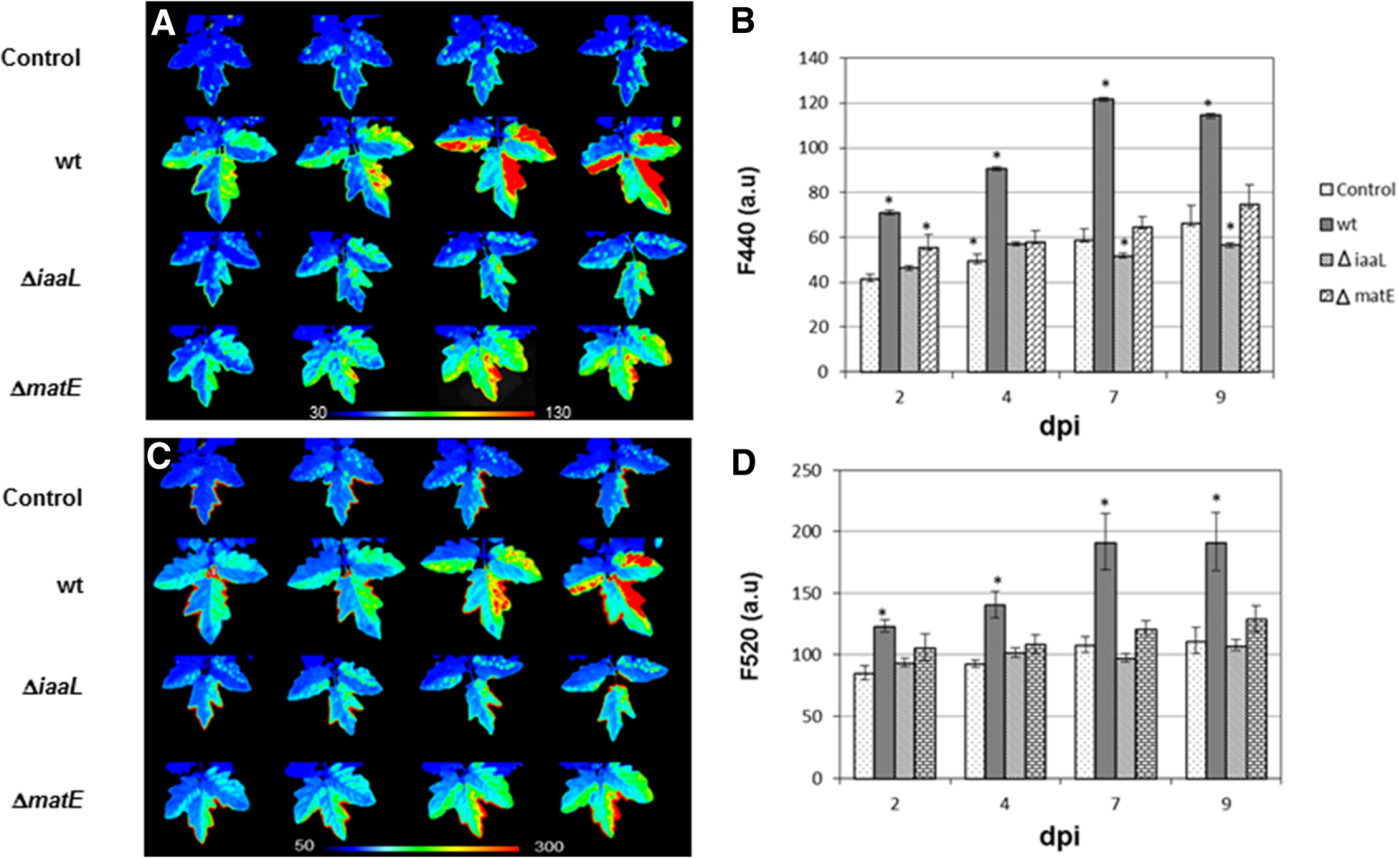
Host response to infection with *P. syringae* pv. tomato DC3000 and its Δ*iaaL* and Δ*matE* mutant derivatives. **a** Images and **b** time course of blue fluorescence emission (F440) from tomato leaves infiltrated with either MgCl_2_ (control plants) or the indicated strains. **c** Images and **d** time course of green fluorescence emission (F520). Pictures show representative images of the fluorescence measurements carried out in control and bacteria-challenged tomato plants at 2, 4, 7 and 9 dpi. Three different experiments with four repetitions for every sample were performed. The *color scale* applied is shown for panels **a** and **c**. Averages for the whole inoculated area at each dpi assayed are displayed; *error bars* indicate standard error and significant differences are marked (*)

Imaging techniques have emerged as valuable tools to investigate the spatial and temporal heterogeneity of plant responses to pathogens [55]. Multicolor fluorescence imaging (MCFI) can reveal leaf patterns of autofluorescence emitted either by chlorophyll or secondary metabolites involved in plant defense (BFG, blue-green fluorescence), allowing pathogen diagnosis in the absence of visual symptoms [56–58] Excitation of leaves with long-wavelength UV radiation results in the emission of characteristic broad fluorescence bands at approximately 440 nm (blue; F440), 520 nm (green; F520), and 690 nm (red; F690). The first two fluorescence bands are emitted by phenolic compounds, most of which accumulate in response to stress, and the latter by chlorophyll [55]. Here, we used MCFI to describe the responses of tomato plants to wild-type Pto DC3000 and its Δ*matE* and Δ*iaaL* mutants. At 2 dpi, a severe increase in F440 (Fig. 5a and b) and F520 (Fig. 5c and d) was observed in the tissues infiltrated with the wild-type strain compared to the corresponding zones of the mock-control leaves. These results suggested the activation of secondary metabolism related to phenolic compounds in response to bacterial infection. Both F440 (Fig. 5a and b) and F520 (Fig. 5c and d) reached very high values in the final stages of infection (7 dpi and, particularly, at 9 dpi), due to tissue necrosis [59]. Leaves infiltrated with either the Δ*matE* or the Δ*iaaL* mutant exhibited the same trend at 2 and 4 dpi; however, F440 and F520 reached lower values than in the case of leaves infected with the wild-type strain (Fig. 5). Our results are in agreement with previous studies reporting an increase in BGF in plants infected by pathogens [38, 56, 57, 60]. Moreover, these results correlate with the reported accumulation of flavonoids and other phenolic compounds in tomato plants infected with Pto DC3000 [61]. Interestingly, and concomitant with the hypovirulent phenotypes demonstrated by the Δ*matE* and Δ*iaaL* mutants (Fig. 4), the observed changes in F440, F520 were strongest in the case of plants challenged by the wild-type strain, indicating lower activation of secondary metabolism in plants infected with the Δ*matE* and Δ*iaaL* mutants (Fig. 5). All of these plant responses were expected for the decreased bacterial populations exhibited by the mutant strains in comparison with the wild-type strain (Fig. 4).

## Conclusions

After the complete genome sequencing of Pto DC3000, the application of several high-throughput experimental screens and bioinformatics approaches allowed the identification of a suite of genes regulated by the sigma factor HrpL in this model bacterial phytopathogen. In addition to the genes required for the biosynthesis of T3SS and its effectors, other non-effector regulon members with diverse functions were also identified, including the *iaaL* gene and the putative MATE family transporter gene here called *matE*. In this work, we show that the *iaaL* gene, which is highly conserved within the *P. syringae* complex, is found in synteny with the *matE* gene in the genome of *P. syringae* and *P. savastanoi* strains belonging to genomospecies 2, 3 and 4. We demonstrate that in Pto DC3000, both of these genes encode upstream *hrp* box-like promoters that are HrpL-dependent. We also present evidence for the cotranscription of *iaaL* and *matE* (*matE*-*iaaL* operon) in this pathogen. Finally, we show that deletion of either the *iaaL* or the *matE* gene in Pto DC3000 results in decreased fitness, colonization and virulence, together with a lower activation of secondary metabolism in infected tomato plants.

## Additional files

Additional file 1: Figure S1. Prediction of transmembrane helices in the MatE protein of *P. syringae* pv. tomato DC3000. Transmembrane regions are shown in red, internal regions in blue, and external regions in pink. The Y-axis shows the probability of the domains being transmembrane, internal or external regions. (TIFF 503 kb) Additional file 2: Figure S2. Determination of the transcription start sites of the *iaaL* and *matE* genes in *P. syringae* pv. tomato DC3000. Total RNA of *P. syringae* pv. tomato DC3000 grown on minimal media was extracted. (**A**) For *iaaL*, runoff cDNAs were generated by extending primers R161 and R121 (Table S1). Lanes A, C, G, and T contain the products of the dideoxy sequencing reactions with using primer R161. Arrows indicate the size of the runoff cDNAs obtained for each primer. (**B**) Nucleotide sequence of the intergenic region including the 3′ and 5′ ends of the *matE* and *iaaL* ORFS, respectively. (**C**) For *matE*, the +1 position was determined by 5′RACE and subsequent sequencing. B and C: The coding sequences of both genes are shadowed. The transcription start sites (P_*iaaL*/matE_) are marked with a black spot and are shown in bold type; the arrowhead indicates the direction of transcription. The *hrp* box motifs are shown in gray boxes, and the sequences of the primers used are underlined. (TIFF 3064 kb) Additional file 3: Figure S3. Distances of the predicted transcriptional start sites of *P. syringae* pv. tomato DC3000 HrpL-regulated genes starting from the 3′ ends of their corresponding *hrp* boxes. Distances were calculated using the 52 annotated *hrp*-boxes (http://www.pseudomonas-syringae.org/) and the 49 transcription start sites previously predicted [34]. (TIFF 164 kb) Additional file 4: Table S1. Primers used in this study. (DOC 57 kb)
