# Supplementary figures and images for: Contribution of the non-effector members of the HrpL regulon, iaaL and matE, to the virulence of Pseudomonas syringae pv. tomato DC3000 in tomato plants
Source: BMC Microbiol. 2015 Aug 19;15:165. doi: 10.1186/s12866-015-0503-8 (PMC4544800; doi:10.1186/s12866-015-0503-8)

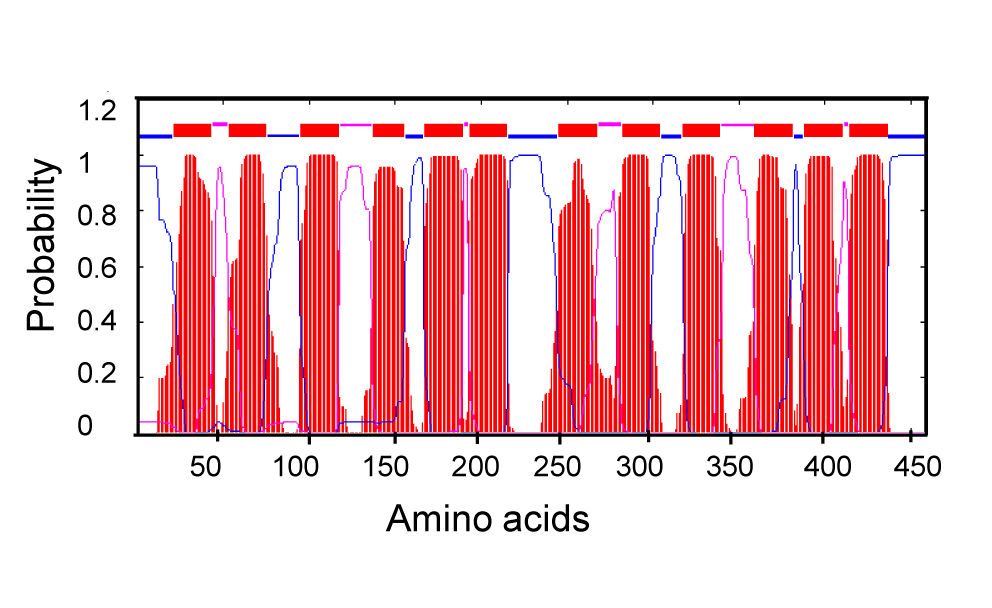

Supplement: Additional file 1: Figure S1. — Prediction of transmembrane helices in the MatE protein of P. syringae pv. tomato DC3000. Transmembrane regions are shown in red, internal regions in blue, and external regions in pink. The Y-axis shows the probability of the domains being transmembrane, internal or external regions. (TIFF 503 kb) [file 12866_2015_503_MOESM1_ESM.tif]

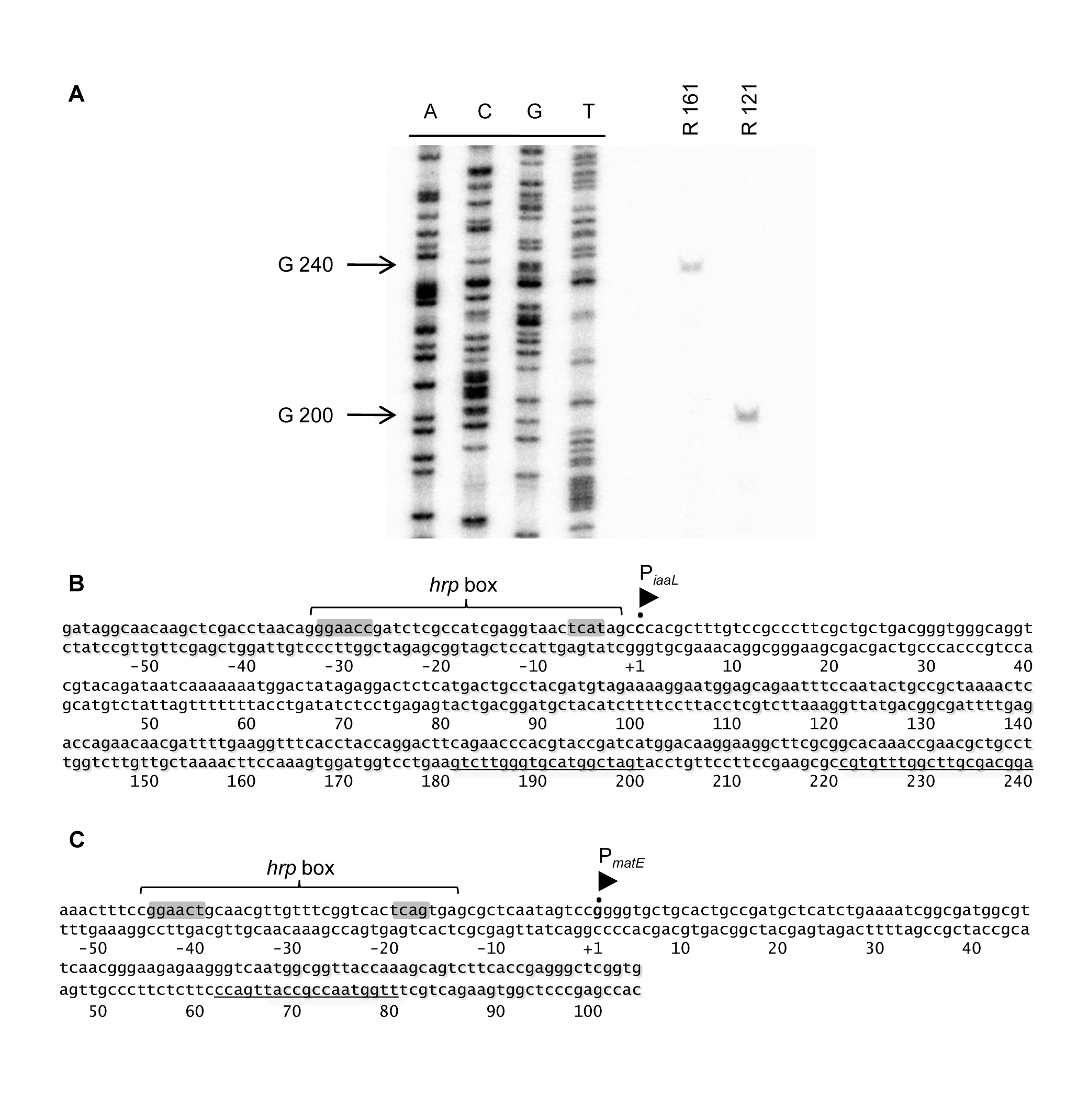

Supplement: Additional file 2: Figure S2. — Determination of the transcription start sites of the iaaL and matE genes in P. syringae pv. tomato DC3000. Total RNA of P. syringae pv. tomato DC3000 grown on minimal media was extracted. (A) For iaaL, runoff cDNAs were generated by extending primers R161 and R121 (Table S1). Lanes A, C, G, and T contain the products of the dideoxy sequencing reactions with using primer R161. Arrows indicate the size of the runoff cDNAs obtained for each primer. (B) Nucleotide sequence of the intergenic region including the 3′ and 5′ ends of the matE and iaaL ORFS, respectively. (C) For matE, the +1 position was determined by 5′RACE and subsequent sequencing. B and C: The coding sequences of both genes are shadowed. The transcription start sites (PiaaL/matE) are marked with a black spot and are shown in bold type; the arrowhead indicates the direction of transcription. The hrp box motifs are shown in gray boxes, and the sequences of the primers used are underlined. (TIFF 3064 kb) [file 12866_2015_503_MOESM2_ESM.tif]

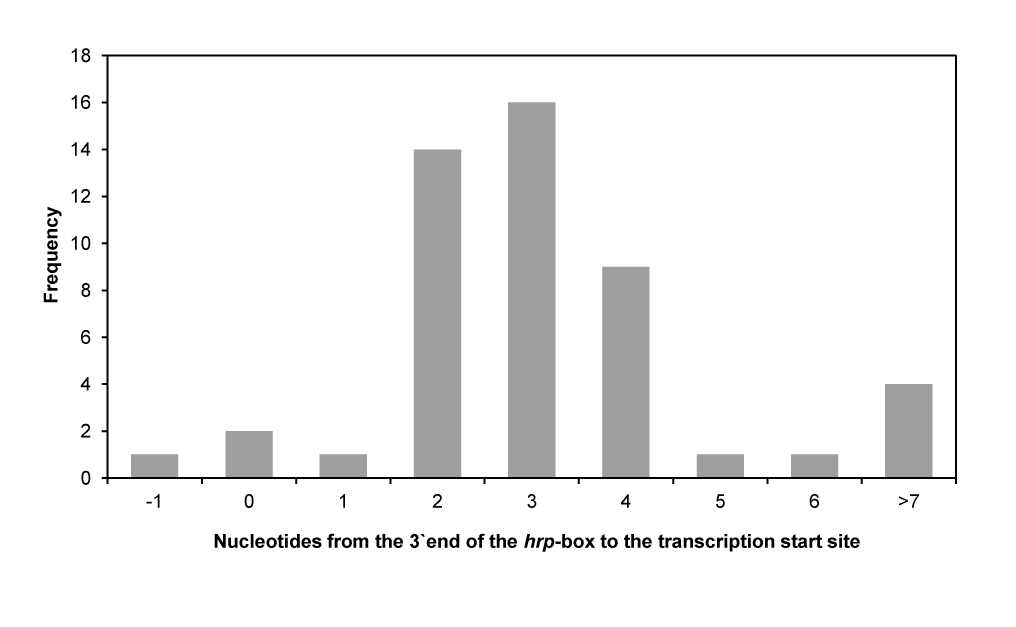

Supplement: Additional file 3: Figure S3. — Distances of the predicted transcriptional start sites of P. syringae pv. tomato DC3000 HrpL-regulated genes starting from the 3′ ends of their corresponding hrp boxes. Distances were calculated using the 52 annotated hrp-boxes (http://www.pseudomonas-syringae.org/) and the 49 transcription start sites previously predicted [34]. (TIFF 164 kb) [file 12866_2015_503_MOESM3_ESM.tif]
