# Supplementary material for: Contribution of the non-effector members of the HrpL regulon, iaaL and matE, to the virulence of Pseudomonas syringae pv. tomato DC3000 in tomato plants
Source: BMC Microbiol. 2015 Aug 19;15:165. doi: 10.1186/s12866-015-0503-8 (PMC4544800; doi:10.1186/s12866-015-0503-8)
Supplement: Additional file 4: Table S1. — Primers used in this study. (DOC 57 kb) [file 12866_2015_503_MOESM4_ESM.doc]

**Table S1. Primers used in this study.**

| **Primer name** | **Sequence 5´-3´** | **Restriction Site*** | | **Application** |
| --- | --- | --- | --- | --- |
| Porina F-1172 | ACCGATCAGAAGCGTTGG | NA | *matE* mutant | |
| TA *matE* R-14 | CCCTATAGTGAGTCGGATCCCCATTGACCCTTCTCTTC | BamHI | *matE* mutant | |
| *matE* F-1338 | GGATCCGACTCACTATAGGGGGATAGGCAACAAGCTCGA | BamHI | *matE* mutant | |
| *iaaL* R-611 | ACGATGGAGACCTGTTGC | NA | *matE* mutant | |
| *matE* F-944 | CAACCACCTCGCTGTTGC | NA | *iaaL* mutant | |
| TA *iaaL* R-80 | CCCTATAGTGAGTCGGATCCTACATCGTAGGCAGTCATG | BamHI | *iaaL* mutant | |
| TD *iaaL* F-18 | GGATCCGACTCACTATAGGGTGACTGCCTACGATGTAG | BamHI | *iaaL* mutant | |
| TD *iaaL* R-491 | TCGATGAACTGCAGGATG | NA | *iaaL* mutant | |
| *hrpJ* Pto F-314 | TTCAGATCTGAGGAAGAG | NA | *hrpL* mutant | |
| TA *hrpL* Pto R-231 | CCCTATAGTGAGTCGGATCCGAAACATGGGCTTACCCTG | BamHI | *hrpL* mutant | |
| TD *hrpL* Pto F-3 | GGATCCGACTCACTATAGGGCCCGTTCGCCTGAATGG | BamHI | *hrpL* mutant | |
| *hrpK* Pto R-408 | AGAGCTTCTGGCGAGGAG | NA | *hrpL* mutant | |
| P1 | GTGTAGGCTGGAGCTGCTTC | NA | Kanamycin gene | |
| Km R-768 | TTGCATCAGCCATGATGG | NA | Kanamycin gene | |
| *iaaL* R-161 | AGGCAGCGTTCGGTTTGTGC | NA | *iaaL* primer extension | |
| *iaaL* R-121 | TGATCGGTACGTGGGTTCTG | NA | *iaaL* primer extension | |
| TA *matE* Pto R-5 | TTGGTAACCGCCATTGACC | NA | *matE* 5´RACE | |
| Porina Pto F-1296 | CCAAGAATTCGTTGAGCGTGCTCTGACC | EcoRI | Promotor PmatE | |
| TD *matE* Pto R-15 | CCAACTGCAGCATTGACCCTTCTCTTCC | PstI | Promotor PmatE | |
| *matE* Pto F-11 | CAACCAAGCTTCTGCCGATGCTCATCTG | HindIII | Promotor PiaaL | |
| *matE* Pto R-1365 | CAACCAAGCTTCTCTCCTATGAGTTACC | HindIII | Promotor PiaaL | |
| *iaaL* F-221 | GGCACCAGCGGCAACATCAA | NA | *iaaL* Northern | |
| *iaaL* R-696 | CGCCCTCGGAACTGCCATAC | NA | *iaaL* Northern | |
| *matE* 1/2 F-237 | TTCGAGAAGCCTGGGCAG | NA | *matE* Northern | |
| *matE* 1/2 R-780 | AGCCATTACGTAGGCCATGC | NA | *matE* Northern | |
| *iaaL* F-283 | CTCCCTCTCCAACGTCTTC | NA | *iaaL* RT-PCR | |
| *iaaL* R-583 | GCCTGATGATTTTCTTCTG | NA | *iaaL* RT-PCR | |
| *matE* F-65 | GCATGTTGTGTTCATGACCAG | NA | *matE* RT-PCR | |
| *matE* R-451 | GATCTGCACGCATAGCTGCTC | NA | *matE* RT-PCR | |
| *matE* F-944 | CAACCACCTCGCTGTTGC | NA | intergenic *matE-iaaL* RT-PCR | |
| *iaaL* R-141 | AGGCAGCGTTCGGTTTGTGC | NA | intergenic *matE-iaaL* RT-PCR | |
| *matE* 1/2 F-1191 | ACCATGCCGTTCGTGTGG | NA | intergenic *matE-iaaL* RT-PCR | |
| *iaaL*1/2 R-265 | CTTCATCGACTTCGCAGTCGTAG | NA | intergenic *matE-iaaL* RT-PCR | |
| TA *iaaL* Pto F-32 | GGTACCGTGGGCAGGTCGTACAG | KpnI | pJB3-i*aaL* construction | |
| *iaaL* Pto R-1186 | GAGCTCGCTTTCACATCAGCCATTC | SacI | pJB3-i*aaL* construction | |
| TA *matE* Pto F-46 | GGTACCGGTGCTGCACTGCCGATG | KpnI | pJB3-*matE* construction | |
| TD *matE* Pto R-32 | GAGCTCTCTGTACGACCTGCCCAC | SacI | pJB3-*matE* construction | |

*NA, not applicable.
